# Tall cell variant of papillary thyroid carcinoma: current evidence on clinicopathologic features and molecular biology

**DOI:** 10.18632/oncotarget.8215

**Published:** 2016-03-20

**Authors:** Xiaofei Wang, Wenli Cheng, Chongqing Liu, Jingdong Li

**Affiliations:** ^1^ Department of General Surgery, Affiliated Hospital of North Sichuan Medical College, Nanchong, China; ^2^ Department of Otolaryngology-Head and Neck Surgery, Affiliated Hospital of North Sichuan Medical College, Nanchong, China

**Keywords:** thyroid cancer, papillary carcinoma, tall cell, molecular biology, review

## Abstract

Tall cell variant (TCV) of papillary thyroid carcinoma (PTC) has been recognized for the past few decades as an entity showing aggressive biological behavior; however, there is considerable controversy regarding the definition, clinical and pathological features of TCV because of its rarity and difficult diagnosis. No clinical features can accurately diagnose TCV. Thus, the results of histocytology, immunohistochemistry and molecular genetics tests have important clinical implications for diagnosis. Given the aggressiveness and the increased recurrence and poor survival rates, more aggressive treatment approach and rigorous follow-up is required for patients with TCV. In the present article, we undertook a comprehensive review to summarize and discuss the various aspects of this variant, from morphology to immunohistochemistry, and molecular abnormalities from a practical and daily practice-oriented point of view.

## INTRODUCTION

Although most of papillary thyroid carcinoma (PTC) are indolent with excellent long-term survival, several histological variants of PTC have been found to be associated with more aggressive biologic behavior and poorer prognosis [1, 2]. Tall cell variant (TCV), the most common aggressive variant of PTC, frequently has vascular invasion, extrathyroidal extension, lymph node metastasis, and distant metastases [3–9], and its 10-year overall and disease-free survival has been reported to be approximately 10% lower than that of classic PTC (cPTC) [7]. Considering the aggressive clinical features and high mortality, a number of studies on the characteristics and prognosis of TCV have been reported over the last two decades. However, most of the information about this subtype was based on small single institution retrospective studies due to its rarity and difficult diagnosis. Along with increased reported cases of TCV, there is considerable debate regarding the clinical and pathological features of TCV. In order to clarify these issues, we undertook a review to summarize and discuss the clinical, pathological and molecular features of this variant.

## DEFINITION

TCV was first described and defined as a papillary thyroid cancer composed of at least 30 % tall cells by Hawk and Hazard in 1976 [10]. The tall cell is characterized by a height greater than twice its width, eosinophilic cytoplasm, basilar oriented nuclei, besides the classic nuclear features of PTC [11–13]. The 2004 World Health Organization (WHO) classification defined a PTC as TCV if it contained 50% or more tall cells [14]. However, there is controversy with regard to the proportion of tall cell required for diagnosis of TCV in literature. The threshold of this proportion varies from 10% to 75% across these studies with > 30% being the most commonly used threshold (*n* = 10 studies) [6, 7, 13, 15–21], followed by > 50% (*n* = 7 studies) [3, 8, 22–26].

The percentage of tall cell necessary to diagnose this variant is various. In one study, Ganly et al [4] found that PTC cases containing 30%-49% tall cells and cases containing ≥ 50% tall cells have similar clinicopathologic features and survival, but both of them bear more aggressive features and poorer prognosis than PTC cases with < 30% tall cells. Therefore, they recommended the threshold of tall cells in the tumor for the diagnosis of TCV should be set at 30%. However, two other studies [27, 28] found that PTC with ≥10% tall cells demonstrated more aggressive clinicopathologic features than cPTC, and they suggested the presence of more than 10% tall cells in a tumor should be reported on final pathology reports. Considering the controversial definition on TCV at present, it is rational that the presence of foci of tall cells should be mentioned in pathologic reports irrespective of percentage of tall cell cytology found as suggested by LiVolsi VA [29]. Further studies need to be done to determine the appropriate definition.

## EPIDEMIOLOGY

**Figure 1:**
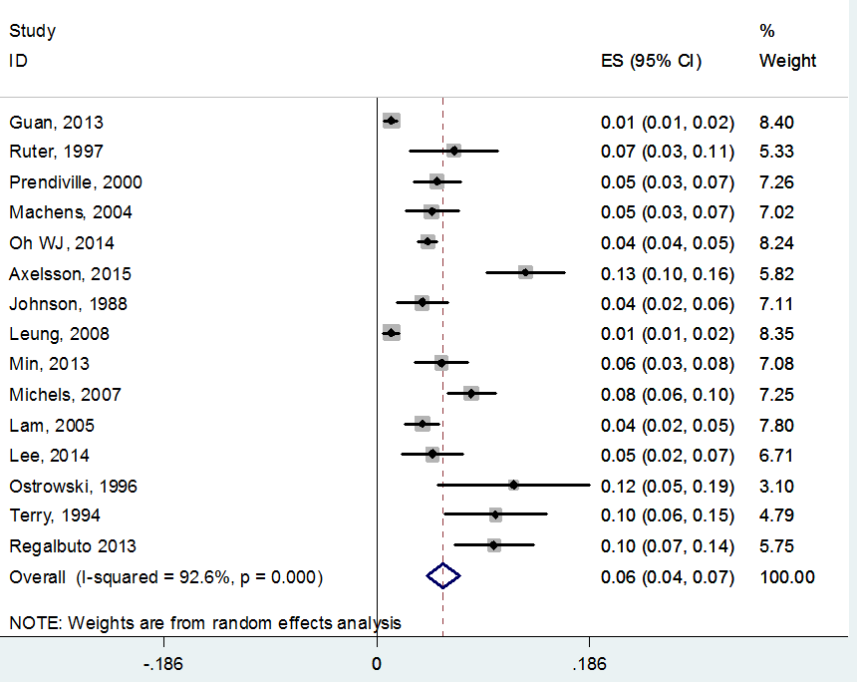
The weighted incidence of TCV pooling the information from the literature

The incidence of TCV varies from 1.3% to 13% of all PTC in literature [3, 18, 21, 24]. The variation in reported prevalence was mainly due to the various diagnostic criteria, difficulty in distinguishing TCV from other variants, and less attention paid to this entity at routine pathological examination. In addition, the incidence of this variant is believed to be underdiagnosed. Several studies [7, 8, 16, 30] have shown that 1-13% of cases diagnosed as cPTC previously were reclassified as TCV when reviewed by endocrine pathologists. After pooling the information from the literature, the weighted incidence of TCV is 6% (95%CI: 4%-7%) (Figure 1).

The incidence of TCV is found to be on the rise with the increasing incidence of PTC worldwide. In one study [31] including 573 TCV and 42,904 cPTC from the Surveillance, Epidemiology, and End Results (SEER) database (1988-2008), authors found the TCV incidence increased by 158%, while the cPTC incidence increased by 60.8%. A similar result was observed in other study [32], which also indicates that TCV incidence has an increase in more recent years. Undoubtedly, a major component of the increased TCV incidence is due to widespread detection of thyroid cancers and more attention on TCV by pathologists. However, similar to the incidence of PTC, whether it is a true increased in TCV is still in controversy, and the reason for this is unknown.

## CLINICAL FEATURES

Similar to cPTC, most studies found that females are predominately affected by TCV. The pooled clinical characteristics of all reported TCV cases and related control patients with cPTC in literature are summarized in Table 1. Of the 843 TCV patients, women accounted for 74% (*n* = 625), and men accounted for 26% (*n* = 218). The female to male ratio was about 2.9 to 1. In the control patients with cPTC, women accounted for 80%, and men accounted for 20%. The female to male ratio was about 4.1 to 1. Moreover, some studies observed a male predominance in patients with TCV [30, 33]. Thus, TCV has a relatively higher prevalence in males than cPTC.

The mean age at presentation of patients with TCV ranges from 41 to 66 years, but the age of cPTC patients ranges from 34 to 53 years in literature. A recent SEER database study [31] also showed that the age at presentation of patient with TCV was older than that of cPTC patients (55.3 years vs. 47.1 years). In addition, another study [34] on SEER database came to similar conclusions. Pooling the data from the reported series so far, the mean age at presentation in patients with TCV and cPTC was 50.1 and 45.7 years respectively (Table 1). Thus, TCV patients tend to be older than those with cPTC at the time of diagnosis.

There is no consensus on whether TCV is different from the cPTC in terms of tumor size, multifocality, lymph node involvement, or distant metastasis at presentation. Pooling of data for this rare variant may answer these questions. As shown in Table 1, the overall rate of multifocality, extrathyroidal extension, lymph node metastasis, and distant metastases at the time of diagnosis in patients with TCV was 45.7%, 63.9%, 59.3% and 8.6% respectively, compared to 32.7%, 33.5%, 33.7% and 3.0% in cPTC patients. The mean tumor diameter in all cases reviewed was 2.0cm in TCV patients and 1.9cm in cPTC patients. Thus, the TCV may be associated with aggressive features when compared to cPTC.

**Table 1:** Pooled data of clinical and pathological features in tall cell and cPTC case series

	Tall cell			cPTC		
	Mean	Range	N	Mean	Range	N
Mean age at diagnosis (y)	50.1	41--66	874	45.7	34--53	2962
Gender (F/M)	2.9	1.0--6.3	843	4.1	1.4--15	3748
Mean tumor size (mm)	20.1	7--42	505	19.0	7--28	2760
Mutifocality (%)	45.7	27.0—61.5	449	32.7	24.1--45	2300
Extrathyroidal extension (%)	63.9	33.3—94.1	631	33.5	0—63.6	2887
Lymph node metastasis (%)	59.3	23.3—83.3	740	33.7	12.5—54.8	3467
Distant metastases (%)	8.6	0—18.2	315	3.0	0—7.1	1947
Recurrence (%)	22.2	0—66.7	442	6.5	0—14.3	1467
Cause-specific death (%)	8.3	1.5—42.9	290	0.03	0—0.10	3229

## PRE-OPERATIVE DIAGNOSIS

Thyroid ultrasound has been widely used to diagnosis of thyroid nodules, and the sonographic features for PTC has been well established, including nodule hypoechogenicity, presence of microcalcifications, irregular margins, and a sharp taller than wide measured on a transverse view [2, 35]. Due to the rarity of TCV, only one study [23] reported the distinctive sonographic appearance of this variant to date. In that study, Choi et al reviewed the sonographic features of 10 TCV and found that TCV always associated with microlobulated, markedly hypoechoic solid nodules, extrathyroidal extension and lymph node metastasis. Ultrasound elastography for thyroid nodules is gaining more attention recently [36, 37], but its diagnostic value in TCV is still unknown. Thus, further studies focused on the sonographic features of TCV are required.

Fine needle aspiration biopsy (FNAB) is a readily available and reliable way to diagnose PTC before surgery. In most cases, TCV tumors exhibited many similar cytologic features to those of cPTC, such as the nuclear grooves and nuclear pseudoinclusions. However, given the aggressiveness of this variant tumor, it would be helpful if this variant is distinguished from cPTC by cytologic characteristics on FNAB prior to surgical treatment. According to the evidence in literature [12, 13, 24, 38], the reliable distinguishing features of TCV from cPTC were large polygonal cells with abundant granular oncocytic cytoplasm, distinct cytoplasmic borders, prominent central nucleoli, and increased numbers of nuclear pseudoinclusions, imparting a “soap bubble” appearance to the nucleus.

## CLINICAL MANAGEMENT

As commented above, TCV tends to occur more frequently in males and older population, with an increased incidence of multifocality, extrathyroidal invasion, lymph node involvement, and distant metastasis at presentation. All of the features associated with worse prognosis point out that patients with TCV should undergo a more aggressive treatment regimen and followed closely. Patients with TCV should be benefit from total thyroidectomy with routine prophylactic central lymph node dissection at initial operation. A therapeutic lateral lymph node dissection should be performed if the lateral lymph nodes involvement is confirmed by preoperative imaging or FNAB. Moreover, because of the high incidence of local nodal involvement and distant metastasis, adjuvant radioactive iodine therapy should be considered for all patients regardless of tumor size. In addition, a more aggressive postoperative thyrotropin suppression therapy and rigorous follow-up should be considered.

## PROGNOSIS

There is a controversy about whether TCV is associated with worse prognosis. It is difficult to perform a well-conducted survival analysis study in a relatively rare disease with a low death rate. Most studies [5, 26, 28, 39] demonstrated TCV as a more aggressive variant of PTC with higher recurrence and poorer survival compared with cPTC. Similarly, a meta-analysis performed with 131 cases concluded that TCV was a negative prognostic indicator in PTC [40]. However, it has been debated that whether the worse prognosis associated with TCV is attributable to various adverse clinico-pathological parameters or histological type alone. Some studies [7, 18, 41] have shown that TCV histology was not an independent risk factor for thyroid cancer recurrence and death postoperatively, and the most relevant factors being age, tumor size, ex­trathyroidal extension, and TNM stage in multivariate analysis.

In contrast, a number of other studies [8, 16, 42] have reported the contrary. They found the histology did independently affect the recurrence and survival on multivariate analysis. Similar results were also reported in one study including 278 TCV patients and 2522 cPTC patients from SEER database. In that study [34], authors found the 5 year disease-specific survival of TCV patients was poorer than all PTC patients (81.9% vs. 97.8%, *P* < 0.01), and it remained poorer (81.9% vs. 91.3%, *P* = 0.049) when compared with matched cPTC patients for age, sex, presence of gross extrathyroidal disease, regional and distant metastatic stage, and so on. Another study [31] based on the SEER database also found that TCV histology was associated with significantly shorter 5-year overall survival (80.6% TCV vs. 93.5% cPTC, *P* < 0.01) after adjustment. It is interesting that a retrospective analysis of 97 TCV and 18260 cPTC from the SEER database (1998-2009), which limited their analysis only to patients with tumor ≤1cm, did not find a significant differences in overall and disease-specific survival between the histologies [43].

By analyzing the pooled data from the literature, the rates of recurrence and cause-specific death were, respectively, 22.2% (98/442) and 8.3% (24/290) in patients with TCV versus 6.5% (96/1467) and 0.03% (84/3229) in patients with cPTC. Patients with TCV experience higher risk of recurrence and death compared to patients with cPTC.

## BIO-MARKERS

In recent years, the BRAF mutation has emerged as a molecular maker related to aggressive tumor behavior, such as tumor size, extrathyroidal extension, multifocality, lymph nodal metastasis, tumor recurrence and advanced stage in PTC [44–47]. Current evidence suggests that the BRAF mutation has a higher prevalence in TCV with reports ranging from 80% to 100% when compared to cPTC with same reports ranging from 63% to 83% [5, 26, 28, 39, 48]. Thus, the higher prevalence of BRAF mutation may play a role in the aggressive biological behavior of the TCV. However, additional data are needed for understanding the molecular basis of this aggressiveness.

Rearranged during transfection/papillary thyroid carcinoma (RET/PTC) gene rearrangements are one of the most frequent genetic alterations found in PTC, which were a particular feature of approximately 20% of PTC [49–51]. Up to now, 13 different RET/PTC oncogenes have been discovered, and RET/PTC1 and RET/PTC3 represent the most common types, accounting for approximately 90% of RET-associated PTC [51, 52]. Only one study compared the occurrence of RET/PTC between TCV and cPTC in literature. In that investigation [19], a striking correlation between the RET/PTC3 rearrangement and the TCV phenotype was found. Only the RET/PTC3 rearrangement was noted in 35.8% (14/39) of the TCV samples, while the RET/PTC3 rearrangement was noted in 17.2% (6/39) and RET/PTC1 in 10.3% (5/39) of the cPTC samples. The higher incidence of RET/PTC3 in TCV compared to the cPTC suggests that molecular pathogenesis in TCV may be different, which need further studies.

Several immunohistochemical markers have been reported, which may explain the aggressive behavior of TCV. c-Met, a tyrosine kinase receptor, has been implicated in inducing tumor cell motility, survival, proliferation, morphogenesis and angiogenesis [53–55]. It has been demonstrated that c-Met overexpression enhanced the motility and invasiveness of PTC cells [56–58]. Subsequently, c-Met was found to have higher expression in TCV specimens than in cPTC specimens [59]. Similar, MUC1 protein, mediating cell-cell adhesion, and Type IV collagenase, promoting tumor invasion and metastasis, were also shown overexpression in TCV compared with PTC [60, 61]. Tumor-suppressor p53 plays a critical role in tumor prevention, whose mutation was found in approximately 50% of all human cancers [62, 63]. In PTC, overexpression of P53 protein was also observed and related to clinicopathologic and prognostic indicators of PTC [64, 65]. Compared to cPTC, there was a higher rate of P53 protein overexpression in the TCV samples and the p53 mutant positivity correlated with increased rate of local and distant recurrence [17]. Together, those results provided a rational basis for to distinguish the aggressive subtype from cPTC at a molecular level.

Although recent studies have provided significant insights into the molecular events underlying cPTC, there is no sufficient research on the mechanism of aggressiveness of TCV. Moreover, in order to provide valuable diagnostic, prognostic, and therapeutic information, further study needs to be done for more precise and specific biomarkers.

## CONCLUSIONS

TCV, the most common of the aggressive variant of PTC, has a 6% (95%CI: 4%-7%) prevalence in all PTC after pooling the data from the literature, and the prevalence has a significant increasing tendency in more recent years. Although some controversy exists, a number of reports here reviewed, including large database studies, suggest that TCV is more aggressive than cPTC, being associated with older age, bigger tumor diameter, and an increased incidence of multifocality, extrathyroidal extension, lymph node involvement, and distant metastases at presentation, more recurrence and cause-specific death. Thus, more aggressive treatment and rigorous follow-up should be considered for patients with TCV. On the other hand, a number of genetic tests or protein expressions for TCV have been investigated; however, the precise and specific biomarkers for TCV diagnosis or prognosis have not been yet identified, which need more studies.

## MATERIALS AND METHODS

All English language literatures that reported features of TCV and published in peer-review journals were identified by searching PubMed and EMbase databases. Studies with five or more TCV cases were included in this analysis. When the same or overlapping cases were reported in multiple publications, the most updated or complete report with the largest sample size was selected. Following this, the demographic and clinical data of those cases was extracted from each literature source using a pretested form and analyzed using the statistical software SPSS version 16.0 (IBM, New York, USA) and Stata 11.0. A total of 24 individual studies [3–8, 13, 15–28, 39, 59, 66] including 874 patients with TCV met the criteria for critical analysis of the clinical data. To avoid duplicate record, three studies [31, 34, 43] based on SEER database were excluded from data analysis, but they were included in related discussion.
